# Zoonotic relevance of multidrug-resistant bacteria in parrots with respiratory illness

**DOI:** 10.1007/s11259-025-10752-6

**Published:** 2025-05-08

**Authors:** Ahmed Samir, Tarek Mosallam, Hassan Aboul-Ella, Aisha Ali, Ojena Samir, Mohamed Hegab, Mark Erian, Fady Youssef, Hala Zaher

**Affiliations:** 1 Department of Microbiology, Faculty of Veterinary Medicine, Cairo University, Cairo, Egypt; 2 Animal Reproduction Research Institute, Giza, Egypt; 3 LeptoVet Veterinary Laboratory, Cairo, Egypt; 4 Pet Vet Clinic, Cairo, Egypt; 5 Rafiki Veterinary Clinic, Cairo, Egypt; 6 Department of Zoonoses, Faculty of Veterinary Medicine, Cairo University, Cairo, Egypt

**Keywords:** Parrots, Respiratory illness, Multidrug-resistant bacteria, Zoonoses

## Abstract

Nowadays, research attention is paid to the investigation of bacterial pathogens in the cloaca of parrots rather than the nasal niche, which is largely ignored. Therefore, this study aimed to investigate the nasal carriage of multidrug-resistant bacteria with zoonotic potential in parrots suffering from respiratory illness. Nasal swabs were collected from 75 sick parrots with respiratory illness, and they were subjected to microbiological isolation and identification, followed by antimicrobial susceptibility testing. *Escherichia coli*, *Klebsiella pneumoniae*, *Proteus mirabilis*, and *Staphylococcus aureus* were isolated with a prevalence rate of 36%, 32%, 26.7%, and 9.3%, respectively, while one isolate (1.3%) of *Staphylococcus pseudointermedius*, *Staphylococcus simulans*, *Staphylococcus sciuri*, and *Enterococcus faecalis* was identified. *E. coli*, *K. pneumoniae*, and *P. mirabilis* were investigated for ESBL genes, *Staphylococcus* species for the *mec*A gene, followed by SCC*mec* typing, and *E. faecalis* for the *vanA* and *vanB* genes. Regarding beta-lactamase-encoding genes, *bla*_TEM_ (97.6%), *bla*_SHV_ (48.8%), and *bla*_CTX−M_ (39%) gene families were detected, while *bla*_OXA_ was not found. Sequencing of *bla*_CTX−M_ in one strain of *E. coli*, *K. pneumoniae*, and *P. mirabilis* revealed *bla*_CTX−M−15_. The *mec*A was determined in three *S. aureus* and one *S. sciuri* strain, and the SCC*mec* typing of three MRSA isolates yielded type V, whereas type I in *S. sciuri*. Only the *vanA* gene was recognized in the *E. faecalis* strain. Moreover, 67.1% of bacterial isolates exhibited multidrug resistance. These findings highlight the potential role of parrots in the transmission of multidrug-resistant zoonotic bacteria, which may pose a threat to human contacts.

**Supplementary Information:**

The online version contains supplementary material available at 10.1007/s11259-025-10752-6.

## Introduction

Pet birds, the third most frequent companion animal after dogs and cats, are considered intimate pets of people and play an essential part in their lives (Cong et al. 2014). The majority of caged birds belong to two orders: Passeriformes, which includes canaries and finches, and Psittaciformes, which comprises parrots, parakeets, and lovebirds (Boseret et al. 2013). Parrots are among the most valuable bird species worldwide (Becker Saidenberg et al. 2012). While parrots become infected with several pathogens of public health relevance, these infections are largely ignored compared to zoonoses of other companion animals (Ahmed et al. 2021; Samir et al. 2021). Many zoonotic pathogens are frequently isolated in passerines and parrots, including *Enterobacter* spp., *Klebsiella* spp., *Escherichia coli*, *Serratia* spp. (Hidasi et al. 2013), *Salmonella* spp. (Allgayer et al. 2008), *Yersinia* spp. (Galosi et al. 2015), and *Staphylococcus aureus* (Hermans et al. 2000). Among Enterobacterales, pathogenic *E. coli* can induce both intestinal and extra-intestinal infections (Pokharel et al. 2023). The presence of *E. coli* strains in the guts of psittacine birds is a cause for concern owing to their risk of sepsis and mortality, as well as the possibility of transmission to human contacts (Gioia-Di Chiacchio et al. 2016; Ahmed et al. 2021). *E. coli* is responsible for nosocomial infections in humans, including catheter-related urinary tract infections and ventilator-associated pneumonia (VAP) (Sligl et al. 2006). *K. pneumoniae* could be detected in feces of parrots and passerines (Kekeç et al. 2021), but this pathogen is frequently regarded as a respiratory pathogen, especially in immunocompromised and stressed birds (Davies et al. 2022). In humans, *K. pneumoniae* is an opportunistic pathogen, causing both community-acquired and nosocomial infections (Kang et al. 2006), as well as UTIs, pneumonia, meningitis, sepsis, and pyogenic liver abcess (Abbas et al. 2024). Among psittacine species, *Salmonella* Typhimurium is a commonly isolated *Salmonella* serotype (Georgiades and Iordanidis 2002), with outbreaks having been reported among birds (Ward et al. 2003). The pathogenicity of *Salmonella* is attributed to an array of virulence genes that are associated with clinical manifestations of *Salmonella* infection (Ghoneim et al. 2017). *P. mirabilis* has been observed in pet birds (Machado et al. 2018; Marques et al. 2021), and it is the most widespread *Proteus* species associated with nosocomial infections and multidrug resistance (Chinnam et al. 2021). Furthermore, *P. mirabilis* causes food poisoning (Gong et al. 2019) and extra-intestinal infections in people, mainly UTIs and others such as skin, wound infections, bloodstream infection, meningeoencephalitis, and osteomyelitis (Girlich et al. 2020). Regarding Gram-positive bacteria, *S. aureus* is an opportunistic pathogen that induces omphalitis, bumblefoot, infected hocks, and stifle joints in birds (Hermans et al. 2000; Ahmed et al. 2021; Szafraniec et al. 2022; Royal et al. 2024). This bacterium lives on the skin and mucosa of healthy people; however, it is responsible for a wide range of life-threatening illnesses, such as food poisoning, skin disorders, and respiratory infections in humans (Otto 2014).

A concern regarding the aforementioned pathogens is their resistance to various antimicrobials, complicating the treatment of these infections in birds, and potentially in humans if transmitted through animal contact (Ajayi et al. 2024). Antimicrobial resistance is a multifaceted phenomenon that poses a public health threat worldwide (Prestinaci et al. 2015). Household pets and companion animals are identified as a significant source of multidrug-resistant zoonotic pathogens (Samir et al. 2022, 2024; Jin et al. 2023; Shaker et al. 2025). Of particular concern in enterobacteria is resistance to cephalosporins, which is mediated by extended-spectrum beta-lactamase (ESBL) genes carried by transferable plasmids (Chen et al. 2019; Husna et al. 2023). Also, methicillin-resistant *Staphylococcus aureus* (MRSA) is a superbug pathogen in human and veterinary medicine (Kasela et al. 2023), and multidrug resistance in MRSA is a main therapeutic challenge worldwide (Chew et al. 2023). Bacterial pathogens isolated from parrot cloaca are a common research focus (Sigirci et al. 2020; Kekeç et al. 2021; Marques et al. 2021). However, little is known regarding the nasal carriage of zoonotic pathogens in parrots suffering from respiratory illness, which is transmitted mainly by droplets and aerosols, posing a risk for human contacts. Thus, the main purpose of this study was to investigate the occurrence of multidrug-resistant bacteria with zoonotic potential in parrots suffering from respiratory illness.

## Materials and methods

### Samples collection

Nasal swabs were collected from 75 clinically sick parrots (blue-and-yellow macaws (*Ara ararauna*)) with respiratory manifestations (sneezing, coughing, dyspnea, noisy breathing, and nasal discharges) at veterinary pet clinics in Cairo, Egypt, during the period from November 2023 to April 2024. The samples were obtained immediately after the birds were admitted to the clinic by a skilled pet bird expert veterinarian, placed in Cary-Blair transport medium tubes, and transported to the LeptoVet^®^ laboratory (an ISO 9001/2015 accredited veterinary laboratory in Egypt) for microbiological examination.

## Bacteriological isolation and identification

Each swab was directly plated on the following set of bacteriological plates: blood agar, MacConkey agar, and mannitol salt agar at 37 °C for 24 h, followed by subculturing at 37 °C for 24 h to obtain a single pure colony. After that, the outcome growth of every sample was evaluated separately in each culture. Bacteriological identification was carried out through Gram staining and conventional biochemical tests, which varied according to the retrieved bacterial species. Oxidase, catalase, indole, methyl red, voges-proskauer (VP), citrate, triple sugar iron test (TSI), and urease were the set of tests used to differentiate Enterobacterales members (Green and Goldman 2021). The recovered enterobacteria were *E. coli*, *Klebsiella* spp., and *Proteus* spp. The indole test was applied to distinguish between *P. mirabilis* and *P. vulgaris* (Quinn et al. 2002; El-Tarabili et al. 2022). The coagulase test was used for differentiation of *Staphylococcus* spp. (Sperber and Tatini 1975), while the catalase test, esculin hydrolysis test, and tryptic soy broth with 6.5% NaCl were used to identify *Enterococcus* spp. (Mariam 2021). The growth characteristics and biochemical properties of isolated bacterial species are inserted in supplementary file 1.

## DNA extraction

DNA extraction was performed in the Microbiology Department, Faculty of Veterinary Medicine, Cairo University. Genomic DNA was extracted from all bacterial isolates using the boiling method according to the protocol described by Mudenda et al. (2023), and the extracted DNA was stored at −20 °C for further molecular analysis.

### Molecular confirmation of *Klebsiella pneumoniae* and *Proteus mirabilis* isolates

All suspected *Klebsiella* isolates were screened for the *gyrA* gene, which targets the *Klebsiella* genus, as previously mentioned by Ebomah and Okoh (2020) and Fadare et al. (2023). Thereafter, all genus-confirmed *Klebsiella* isolates were investigated for *K. pneumoniae* using species-specific primers targeting the 16–23 S ITS gene (Turton et al. 2010; Hasani et al. 2020; Fadare et al. 2023). Molecular confirmation of *P. mirabilis* in presumptive *P. mirabilis* isolates was performed using the species-specific primers targeting the *ureR* gene, as detailed by Chinnam et al. 2021.

### Molecular detection of the *Staphylococcus* 16S rRNA gene and partial sequencing

Three *Staphylococcus* isolates that were negative for the *S. aureus nuc* gene were examined for the *Staphylococcus* genus-specific 16S rRNA gene according to Zhang et al. (2004), followed by partial sequencing of this gene to identify *Staphylococcus* species. The partial *Staphylococcus* 16S rRNA sequences retrieved in the present study were *Staphylococcus simulans* (accession no. PQ117066), *Staphylococcus pseudointermedius* (accession no. PQ117734), and *Staphylococcus sciuri* (accession no. PQ117766).

### Antimicrobial susceptibility testing (AST) of bacterial pathogens retrieved in this study

The antimicrobial susceptibility testing of 27 *E. coli*, 24 *K. pneumoniae*, 20 *P. mirabilis*, 7 *S. aureus*, one *S. pseudointermedius*, one *S. sciuri*, one *S. simulans*, and one *E. faecalis* was performed according to the standard Kirby-Bauer disc diffusion method. Antimicrobials were selected from the respective table of agents that should be considered for testing against each bacteria, and the outcome results were evaluated and interpreted according to the Clinical and Laboratory Standards Institute guidelines (CLSI 2021). The antimicrobials involved were: penicillin (P), ampicillin (AMP), cefotaxime (CTX), ceftazidime (CAZ), cefazolin (CZ), cefoxitin (CX), cefepime (CPM), aztreonam (AT), ertapenem (ET), meropenem (MRP), fosfomycin (FO), gentamicin (GEN), amikacin (AK), azithromycin (AZM), tetracycline (TE), doxycycline (DO), ciprofloxacin (CIP), norfloxacin (NX), trimethoprim-sulfamethoxazole (COT), chloramphenicol (C), nitrofurantoin (NIT), vancomycin (VA), erythromycin (E), clindamycin (CD), rifampin (RIF), linezolid (LZ), and quinupristin-dalfopristin (RP). Multidrug-resistant bacterial strains were identified by resistance to at least one agent in three or more antimicrobial categories (Magiorakos et al. 2012).

### Phenotypic identification of ESBL-producing *E. coli*, *K. pneumoniae*, and *P. mirabilis*, as well as methicillin-resistant staphylococci and vancomycin-resistant enterococci

Seventy-one Enterobacterales strains (27 *E. coli*, 24 *K. pneumoniae*, and 20 *P. mirabilis*) were subjected to ESBL phenotypic identification by double-disc approximation test using both cefotaxime and ceftazidime, alone and in combination with clavulanic acid (ceftazidime-clavulanate and cefotaxime-clavulanate) according to CLSI (2021). Ten *Staphylococcus* isolates and one *Enterococcus* strain were tested for cefoxitin and vancomycin resistance, respectively, following CLSI recommendations (CLSI 2021).

### Molecular identification of beta-lactamase-encoding genes in *E. coli*, *K. pneumoniae*, and *P. mirabilis*

The beta-lactamase-encoding genes (*bla*_TEM_, *bla*_SHV_, *bla*_CTX−M_, and *bla*_OXA_) were investigated in 18 *E. coli*, 13 *K. pneumoniae*, and 10 *P. mirabilis* isolates, which showed phenotypic ESBL production using a multiplex PCR protocol described by Fang et al. 2008.

### Partial sequencing of the *bla*_CTX−M_ gene of *E. coli*, *K. pneumoniae*, and *P. mirabilis* strains

Three PCR products from the aforementioned multiplex PCR of multidrug-resistant ESBL-producing *E. coli*, *K. pneumoniae*, and *P. mirabilis bla*_CTX−M_ gene were selected and purified via a QIAquick purification kit (Qiagen, Hilden, Germany). Afterwards, sequencing was carried out using the Big Dye Terminator V3.1 kit (Thermo Fisher, USA) in an ABI 3500 Genetic Analyzer (Applied Biosystems, USA). The three partial sequences of *E. coli*, *K. pneumoniae*, and *P. mirabilis bla*_CTX−M−15_ were deposited in the GenBank under the following accession numbers: PQ144883, PQ144882, and PQ144884, respectively.

### Molecular identification of *S. aureus*, *mec*A gene, SCC*mec* types, *E. faecalis*,* E. faecium*, and *vanA*/*vanB* genes

PCR amplification of the *S. aureus nuc* gene was investigated in ten *Staphylococcus* isolates, according to McClure et al. (2017) and then the *mec*A gene was investigated in four cefoxitin-resistant staphylococci. Afterwards, a multiplex PCR assay was conducted to characterize staphylococcal cassette chromosome *mec* (SCC*mec*) types I to V in four positive *mec*A *Staphylococcus* isolates, as mentioned by Moosavian et al. (2017). The vancomycin-resistant *Enterococcus* isolate was employed in a quadruplex PCR targeting *E. faecalis*, *E. faecium*, *vanA*, and *vanB* genes, as reported by Kariyama et al. 2000.

### Partial sequencing of the *S. aureus mec*A gene

Sequencing of one purified PCR amplicon of the *S. aureus mec*A gene was carried out in an ABI 3500 Genetic Analyzer (Applied Biosystems). The GenBank accession number of the partial *S. aureus mec*A sequence generated in this study was PQ144885.

## Results

### Occurrence of bacterial species retrieved from diseased parrots with respiratory illness

**Fig. 1:**
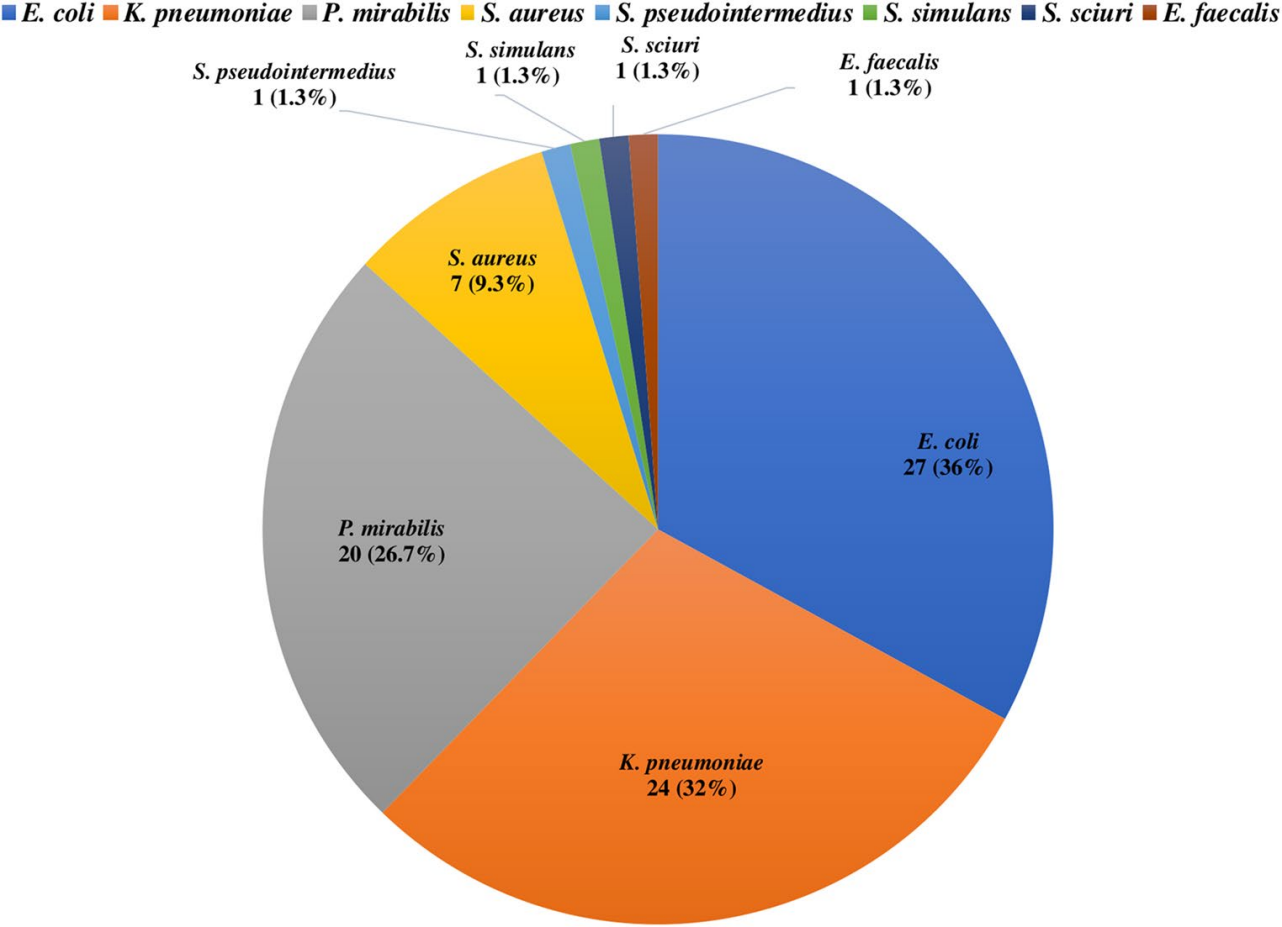
Occurrence of bacterial species isolated from parrots with respiratory illness

Overall, out of 75 diseased parrots, 27 *E. coli* (36%), 24 *K. pneumoniae* (32%), 20 *P. mirabilis* (26.7%), 7 *S. aureus* (9.3%), and one isolate (1.3%) of *S. pseudointermedius*, *S. simulans*, *S. sciuri*, and *E. faecalis* were identified (Fig. 1). The bacterial species retrieved from each parrot are displayed in supplementary file 2.

### Antimicrobial susceptibility pattern of bacterial isolates obtained in this study

**Fig. 2:**
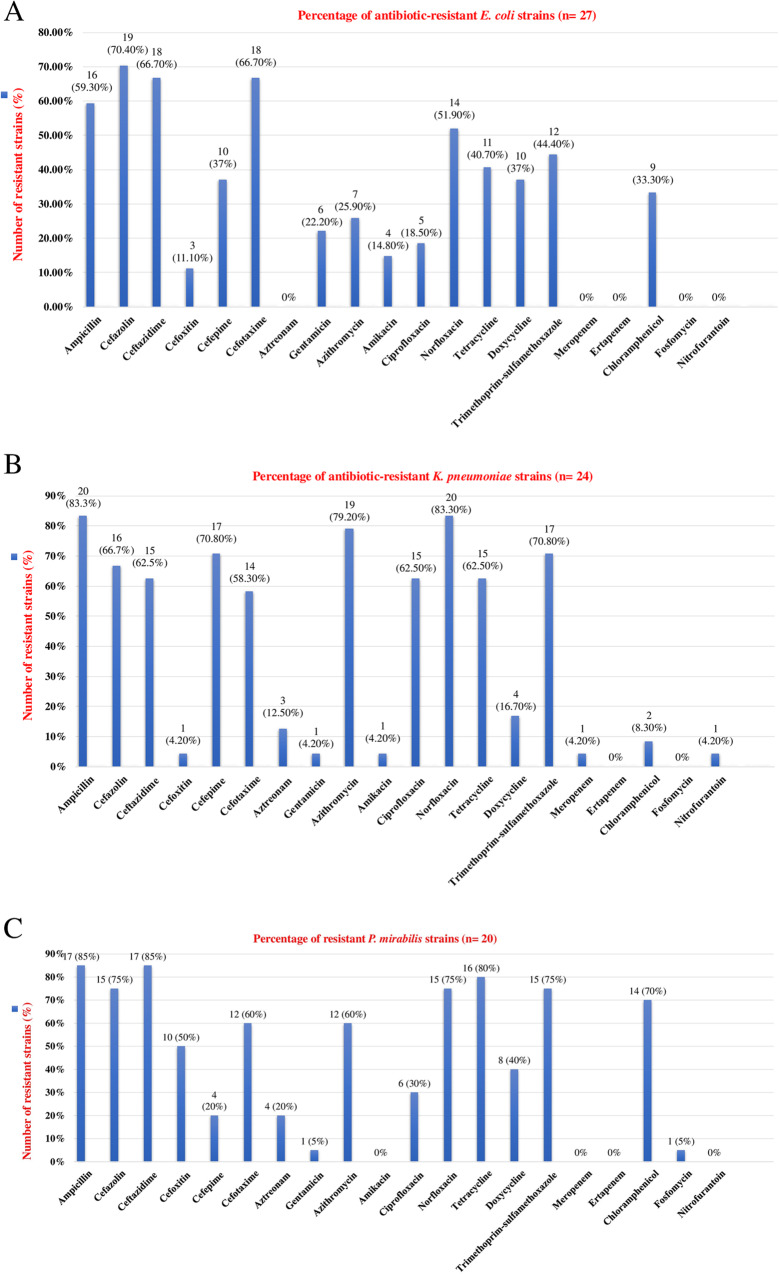
**A** Percentage of antibiotic-resistant *E. coli* strains (*n* = 27). **B** Percentage of antibiotic-resistant *K. pneumoniae* strains (*n* = 24). **C**. Percentage of antibiotic-resistant *P. mirabilis* strains (*n* = 20)

*E. coli*, *K. pneumoniae*, and *P. mirabilis* exhibited high resistance rates to cefazolin (70.4%, 67%, 75%), tetracycline (40.7%, 62.5%, 80%), trimethoprim-sulfamethoxazole (44.4%, 70.8%, 75%), and norfloxacin (51.9%, 83.3%, 75%), respectively, but low resistance to amikacin (14.8%, 4.2%, 0%), gentamicin (22.2%, 4.2%, 5%), and aztreonam (0%, 12.5%, 20%), respectively. All three pathogens were susceptible to ertapenem, as shown in Figs. 2A, B, and C. All seven *S. aureus* isolates showed resistance to norfloxacin and penicillin. One *S. sciuri* isolate displayed resistance to penicillin, cefoxitin, norfloxacin, and clindamycin, whereas the *S. pseudointermedius* and *S. simulans* strains were resistant to clindamycin (Table 1). The resistance profile of the *E. faecalis* strain was presented in Table 1. Multidrug resistance was detected in 83.3% (20/24) of *K. pneumoniae*, 75% (15/20) of *P. mirabilis*, and 55.6% (15/27) of *E. coli* strains. Additionally, three *S. aureus*, one *S. sciuri*, and one *E. faecalis* showed multidrug resistance. The resistance profiles of individual bacterial isolates are presented in supplementary files 3, 4, and 5.

**Table 1:** Antimicrobial susceptibility pattern and occurrence of antimicrobial resistance genes in *Staphylococcus* spp. and *E. faecalis* isolated from diseased parrots

Bacterial species	CX	GEN	AZM	E	TE	DO	CIP	NX	NIT	CD	COT	C	RIF	LZ	RP	*P*	VA	FO	Antimicrobial resistance genes
*S. aureus* (Isolate no.1)	R	S	S	R	R	R	S	R	S	R	R	S	S	S	S	R	N/A	N/A	Positive (*mec*A)
*S. aureus* (Isolate no.2)	R	S	R	R	R	R	S	R	S	R	R	S	S	S	S	R	N/A	N/A	Positive (*mec*A)
*S. aureus* (Isolate no.3)	R	R	R	R	R	S	S	R	S	R	R	S	R	S	S	R	N/A	N/A	Positive (*mec*A)
*S. aureus* (Isolate no.4)	S	S	S	S	S	S	S	R	S	S	S	S	S	S	S	R	N/A	N/A	Negative (*mec*A)
*S. aureus* (Isolate no.5)	S	S	S	S	S	S	S	R	S	S	S	S	S	S	S	R	N/A	N/A	Negative (*mec*A)
*S. aureus* (Isolate no.6)	S	S	S	S	S	S	S	R	S	S	S	S	S	S	S	R	N/A	N/A	Negative (*mec*A)
*S. aureus* (Isolate no.7)	S	S	S	S	R	S	S	R	S	S	R	S	S	S	S	R	N/A	N/A	Negative (*mec*A)
*S. simulans* (*n* = 1)	S	S	S	S	S	S	S	S	S	R	S	S	S	S	N/A	S	N/A	N/A	Negative (*mec*A)
*S. pseudointermedius* (*n* = 1)	S	S	S	S	S	S	S	S	S	R	S	S	S	S	N/A	S	N/A	N/A	Negative (*mec*A)
*S. sciuri* (*n* = 1)	R	S	S	S	S	S	S	R	S	R	S	S	S	S	N/A	R	N/A	N/A	Positive (*mec*A)
*E. faecalis* (*n* = 1)	N/A	N/A	N/A	R	R	R	S	R	S	N/A	N/A	S	S	R	R	R	R	R	Positive (*vanA*)

R: Resistant; S: Susceptible; N/A: Not applicable

*P* Penicillin, *CX* cefoxitin, *FO* fosfomycin, *GEN* gentamicin, *AZM* azithromycin, *TE* tetracycline, *DO* doxycycline, *CIP* ciprofloxacin, *NX* norfloxacin, *COT* trimethoprim-sulfamethoxazole, *C* chloramphenicol, *NIT* nitrofurantoin, *VA* vancomycin, *E* erythromycin, *CD* clindamycin, *RIF* rifampin, *LZ* linezolid, *RP* quinupristin-dalfopristin

### Phenotypic and genotypic detection of ESBL-producing *E. coli*, *K. pneumoniae*, and *P. mirabilis* isolates

A ≥ 5 mm increase in a zone diameter for either antimicrobial agent (ceftazidime or cefotaxime) tested in combination with clavulanic acid versus the zone diameter of agent when tasted alone indicates that the isolate is an ESBL producer. ESBL production was detected among 18 *E. coli*, 13 *K. pneumoniae*, and 10 *P. mirabilis* isolates. All tested ESBL producers were screened for beta-lactamase genes. *bla*_TEM_ was the most predominant beta-lactamase gene family in all three pathogens (97.6%), followed by *bla*_SHV_ (48.8%) and *bla*_CTX-M_ (39%). *bla*_OXA_ was not detected, as shown in Table 2. Further characterization was carried out for *bla*_CTX-M_-containing isolates, but not for other ESBL producers, thus the specific gene variant was not determined.

**Table 2:** Detection of beta-lactamase-encoding genes in ESBL-producing *E. coli*, *K. pneumoniae*, and *P. mirabilis*

Bacterial species	No. of isolates	Beta-lactamase-encoding genes			
		*bla*_TEM_	*bla*_SHV_	*bla*_CTX−M_	*bla*_OXA_
*E. coli* (*n* = 18)	3	+	+	+	-
	1	+	+	-	-
	1	+	-	+	-
	13	+	-	-	-
*K. pneumoniae* (*n* = 13)	3	+	+	+	-
	3	+	+	-	-
	1	-	+	-	-
	6	+	-	-	-
*P. mirabilis* (*n* = 10)	9	+	+	+	-
	1	+	-	-	-
Total	41	40/41 (97.6%)	20/41 (48.8%)	16/41 (39%)	0/41 (0%)

### Occurrence of *mec*A gene in methicillin-resistant staphylococci, SCC*mec* types, and *van* genes in vancomycin-resistant *E. faecalis*

The *mec*A gene was detected in four *Staphylococcus* isolates (*S. aureus* = 3, *S. sciuri* = 1) that were phenotypically resistant to cefoxitin, as displayed in Table 1. Staphylococcal SCC*mec* typing revealed that type V was recognized in three methicillin-resistant *S. aureus*, whereas one methicillin-resistant *S. sciuri* carried type I. The *van* genes were investigated in one vancomycin-resistant *E. faecalis*, and only *vanB* was detected (Table 1).

## Discussion

In the current study, *E. coli* was isolated most frequently from the parrots with respiratory illness. In other studies conducted on pet birds, isolation rates of *E. coli* were 37.7% (Sigirci et al. 2020), 13.2% (Marques et al. 2021), and 46.5% (Lopes et al. 2015). *K. pneumoniae* infection in companion animals has been mainly related to dogs and cats (Marques et al. 2019; Chen et al. 2021; Zhang et al. 2022; Hyeon et al. 2023), but little is known about the occurrence of this pathogen in companion birds (Kekeç et al. 2021; Davies et al. 2016, 2022). The isolation rate of *K. pneumoniae* in this study was lower than that reported by Davis et al. 2022 in companion parrots (34.8%); however, it was higher than that detected by Rueanghiran et al. 2019 in psittacine pet birds (8%). Regarding *P. mirabilis*, there is little data available on the prevalence of this pathogen in pet birds. The occurrence of *P. mirabilis* in our study was nearly similar to that isolated from nestling grey-breasted parakeets (26.4%) (Machado et al. 2018), but it was higher than that found in Brazilian pet shop parrots (17.7%) (Marques et al. 2021).

In this study, *E. coli*, *K. pneumoniae*, and *P. mirabilis* exhibited a high level of resistance to medically important antimicrobials, including ampicillin, cephalosporins, norfloxacin, tetracycline, and trimethoprim-sulfamethoxazole. Similarly, Sigirci et al. (2020) reported that 84% and 46% of *E. coli* isolates obtained from companion birds were resistant to tetracycline and sulfamethoxazole/trimethoprim, respectively, and Hidasi et al. (2013) found that 75.58% and 69.19% of *E. coli* strains isolated from parrots were resistant to ampicillin and tetracycline, respectively. Also, Davies et al. (2016) and Marques et al. (2021) revealed that *K. pneumoniae* and *P. mirabilis* strains retrieved from psittacine birds and parrots showed a high resistance to ampicillin (84.3%) and tetracycline (83.3%), respectively. In contrast to the present study, Pontes et al. 2018 determined that *E. coli* strains isolated from captive cockatiels had a high resistance to aminoglycosides (74%) and low resistance to sulfonamide (33%). Variation in sample size, geographic location, and the use of various antimicrobials may account for differences in the prevalence between studies (Kekeç et al. 2021; Marques et al. 2021). Noteworthy, *K. pneumoniae* displayed the highest multidrug resistance, followed by *P. mirabilis* and *E. coli* among infected parrots in this study. Multidrug resistance was previously identified in *E. coli* obtained from pet birds at varying rates: 67% (Sigirci et al. 2020), 59% (Pontes et al. 2018), 55.7% (Horn et al. 2015), and 33.8% (Hidasi et al. 2013). Unfortunately, data about multidrug-resistant *K. pneumoniae* and *P. mirabilis* in companion birds are confined, with 25% of *K. pneumoniae* strains isolated from psittacine birds in Brazil being multidrug-resistant (Davies et al. 2016), which was lower than the percentage reported in this study. The high percentage of multidrug resistance among such pathogens may be influenced by imprudent use of antimicrobials without veterinary supervision, leading to selection and transmission of resistant bacteria, further reducing antimicrobial efficacy (Caneschi et al. 2023). Importantly, ESBL-producing *E. coli*, *K. pneumoniae*, and *P. mirabilis* were identified in the current work. A lot of reports have documented ESBL-producing Enterobacterales in wild birds (Alcalá et al. 2016; Raza et al. 2017; Yuan et al. 2021; Brendecke et al. 2022; Athanasakopoulou et al. 2022; Saeed et al. 2023), but limited information concerning pet birds is found (Yılmaz and Dolar 2017; Sigirci et al. 2020; Davies et al. 2022). In this study, the most prevalent beta-lactamase-encoding gene family was *bla*_TEM_, followed by *bla*_SHV_ and *bla*_CTX-M_. Because not all TEM or SHV variants are ESBLs, it is difficult to determine whether TEM or SHV genes are ESBLs without identifying the specific variant. In the last decade, CTX-M type ESBLs have become the most frequently distributed ESBLs worldwide, which prevail in community-acquired infections (Azzam et al. 2024) and confer resistance to penicillins, extended-spectrum cephalosporins, and monobactams (Cantón et al. 2012). Accordingly, partial sequencing of the *bla*_CTX-M_ gene in one isolate of *E. coli*, *K. pneumoniae*, and *P. mirabilis* was carried out, and *bla*_CTX-M-15_ was retrieved from all three pathogens. *E. coli* carrying *bla*_CTX-M-15_ has emerged globally as an important driver of bloodstream infections and community-acquired UTIs (Cantón et al. 2012); however, we did not determine whether the *E. coli* variants identified here are related to those that commonly cause human infections.

Concerning staphylococci, the occurrence of *S. aureus* in the current study was higher than that detected in pet birds in Bangladesh (4.1%) (Royal et al. 2024). *S. aureus*, the most significant *Staphylococcus* species, causes food poisoning, skin disorders, wound colonization, and respiratory infections (Otto 2014). To the best of our knowledge, *S. simulans*, *S. pseudointermedius*, and *S. sciuri* were identified for the first time in parrots in this study. *S. pseudintermedius* is a major coagulase-positive staphylococci that leads to opportunistic infections in dogs (Haulisah et al. 2022). Transmission of *S. pseudintermedius* between dogs and humans has been documented, resulting in skin, soft tissue, and bloodstream infections in humans (Blondeau et al. 2021). *S. simulans*, a coagulase-negative staphylococci (CoNS), is associated with endocarditis in chickens (Stępień-Pyśniak et al. 2017), and it causes a variety of illnesses in humans, including bacteremia, skin infections, native valve endocarditis, post-surgical osteomyelitis, and UTIs (Males et al. 1985; Vallianou et al. 2008; Shields et al. 2016). *S. sciuri* has drawn more attention in recent years due to its potential for zoonotic transmission (Dakić et al. 2005). The clinical significance of *S. sciuri* in humans appears to be growing because this pathogen is associated with wound infections, endocarditis, peritonitis, septic shock, UTIs, endophthalmitis, and pelvic inflammatory disease (Dakić et al. 2005). As a result, parrots might be regarded as a possible reservoir for zoonotic *Staphylococcus* species, which may pose a threat to human health.

Interestingly, three methicillin-resistant *S. aureus* (MRSA) isolates were recognized in this study. There are few studies on the detection of MRSA in psittacine birds (Briscoe et al. 2008; Zaman et al. 2020). MRSA infections have increased substantially during the last 10–15 years and they are becoming a major cause of nosocomial infections with high morbidity and mortality (González-Vázquez et al. 2024). In addition, one *S. sciuri* isolate carried the *mec*A gene. CoNS belonging to the *Staphylococcus sciuri* group, which includes *S. sciuri*, are particularly important due to their role in the origin, evolution, and spread of the *mec*A gene (Ruiz-Ripa et al. 2020). Since the SCC*mec* element is a known vector for transferring *mec*A between *Staphylococcus* species (Saber et al. 2017), it is critical to determine the SCC*mec* type in the obtained MR isolates. SCC*mec* type V was identified in three MRSA isolates and type I in one MR *S. sciuri*, with type V being associated with community-acquired MRSA (Boye et al. 2007). These findings indicate that pet owners who are frequently exposed to parrots may be at risk of acquiring methicillin-resistant staphylococci (Zaman et al. 2020). Furthermore, multidrug resistance was exhibited in three MRSA isolates and one *S. sciuri* strain. Three MRSA isolates were resistant to penicillin, erythromycin, tetracycline, norfloxacin, clindamycin, and sulfamethazole/trimethoprim in this work. However, different resistance profiles were documented by Zaman et al. (2020); who found that *S. aureus* strains obtained from ornamental birds had the highest resistance rate to oxytetracycline (80%), followed by chloramphenicol (15%), ciprofloxacin (10%), and gentamicin (2.5%), and Royal et al. (2024); who revealed that *S. aureus* isolates retrieved from pet birds were resistant to ampicillin (42.86%), ciprofloxacin (42.86%), gentamicin (28.75%), and tetracycline (28.57%).

On the other hand, the role of pet birds as a reservoir for vancomycin-resistant enterococci (VRE) has been little investigated. In a study conducted by Cabral et al. (2020), 11.9% (15/126) of the examined psittacine birds were positive for *E. faecalis*, with one isolate showing an intermediate level of resistance to vancomycin; however, one vancomycin-resistant *E. faecalis* strain was identified in this study. *E. faecalis* is the main *Enterococcus* species associated with life-threatening infections, such as endocarditis, UTIs, meningitis, and bloodstream infections, and it is a leading cause of multidrug-resistant infections (Sangiorgio et al. 2024). Additionally, *E. faecalis* is associated with amyloid arthropathy in avian species (Steentjes et al. 2002). In this work, VR *E. faecalis* had the *vanA* gene only. In pet dogs and cats, many reports of vancomycin-resistant enterococci have been documented (Shaker et al. 2024), with the *vanA* gene being the most common among *Enterococcus* strains (Herrero et al. 2004; Iseppi et al. 2020). It was noted that the obtained VRE isolate in the current work was multidrug-resistant, where it was resistant to several antimicrobials, including linezolid. Linezolid- and vancomycin-resistant enterococci are uncommon in veterinary medicine; nonetheless, these bacterial isolates have recently been detected in healthy chickens (Ben Yahia et al. 2024) and fish (Abdel-Raheem et al. 2024). To the best of our knowledge, this is the first report of linezolid- and vancomycin-resistant *E. faecalis* in parrots, which is a public health risk since linezolid is the first line of therapy for VRE infections and provides microbiological success in complicated infections (Misiakou et al. 2023). In conclusion, the recent study highlights the nasal carriage of multidrug-resistant zoonotic bacteria among parrots with respiratory illness, indicating that parrots may be a source of antimicrobial-resistant bacteria to pet owners and emphasizing the importance of enhanced global surveillance for pet bird-related antimicrobial resistance. A limitation of the current study is that we did not perform whole genome sequencing (WGS) due to a lack of resources. WGS provides a broader spectrum of genomic information, enabling a more comprehensive characterization of antimicrobial-resistant bacteria.

## Electronic supplementary material

Below is the link to the electronic supplementary material.

Supplementary Material 1

Supplementary Material 2

Supplementary Material 3

Supplementary Material 4

Supplementary Material 5

## Data Availability

All data generated or analyzed during this study are included in this published article. The partial *Staphylococcus* 16S rRNA gene sequences retrieved in the present study were *Staphylococcus simulans* (accession no. PQ117066), *Staphylococcus pseudointermedius* (accession no. PQ117734), and *Staphylococcus sciuri* (accession no. PQ117766). The GenBank accession number for the partial *S. aureus**mec*A gene sequence generated in this study was PQ144885. The three partial sequences of *E. coli*, *K. pneumoniae*, and *P. mirabilis* CTX-M-15 were deposited in the GenBank under the following accession numbers: PQ144883, PQ144882, and PQ144884, respectively.
