# Supplementary material for: Zoonotic relevance of multidrug-resistant bacteria in parrots with respiratory illness
Source: Vet Res Commun. 2025 May 8;49(4):194. doi: 10.1007/s11259-025-10752-6 (PMC12062053; doi:10.1007/s11259-025-10752-6)
Supplement: Supplementary file 1 — Supplementary Material 1 [file 11259_2025_10752_MOESM1_ESM.docx]

**The growth characteristics and biochemical properties of bacteria isolated from parrots with respiratory illness**

**1-*Escherichia coli***

| **MacConkey agar** | Lactose-fermenting pink colonies |
| --- | --- |
| **Eosin Methylene blue (EMB) agar** | Green metallic sheen |
| **Methyl Red test** | Diffuse red color (positive) |
| **Indole test** | Formation of red ring indicating positive reaction |
| **Voges-Proskauer test** | Negative |
| **Citrate test** | Negative |
| **Urease test** | Negative |
| **Triple sugar iron agar (TSI) test** | Yellow slant and butt with gas but no hydrogen sulfide production (Y/Y/ H_2_S -ve) |
| **Catalase** | Positive (presence of bubbles) |
| **Oxidase** | Negative |

**2-*Klebsiella pneumoniae***

| **MacConkey agar** | Large, mucoid, and pink in color |
| --- | --- |
| **Eosin Methylene blue (EMB) agar** | Mucoid and pink to purple in color with a dark center |
| **Methyl Red test** | Negative |
| **Indole test** | Negative |
| **Voges-Proskauer test** | Change to red color (positive) |
| **Citrate test** | Change to blue color (Positive ) |
| **Urease test** | Change to pink color (positive) |
| **Triple sugar iron agar (TSI) test** | Yellow slant and butt with gas but no hydrogen sulfide production (Y/Y/ H_2_S -ve) |
| **Catalase** | Positive (presence of bubbles) |
| **Oxidase** | Negative |

**2-*Proteus mirabilis***

| **MacConkey agar** | Non-lactose fermenting colorless colonies |
| --- | --- |
| **Blood agar** | Swarming growth |
| **Methyl Red test** | Change to red color (Positive) |
| **Indole test** | Negative |
| **Voges-Proskauer test** | Negative |
| **Citrate test** | Change to blue color (Positive ) |
| **Urease test** | Change to pink color (positive) |
| **Triple sugar iron agar (TSI) test** | Red slant, yellow butt, gas production and H_2_S producer |
| **Catalase** | Positive (presence of bubbles) |
| **Oxidase** | Negative |

**4-*Staphylococcus* species**

| ***Staphylococcus* spp.** | **Mannitol salt agar (MSA)** | **Blood agar** | **Catalase test** | **Coagulase test** |
| --- | --- | --- | --- | --- |
| *S. aureus* | Fermentation of mannitol (yellow colonies on MSA surrounded by yellow zone) | Round, raised, yellow to golden yellow colonies surrounded by zones of clear beta-hemolysis | Positive | Positive |
| *S. pseudointermedius* | Fermentation of mannitol (yellow color) | Round, grey-to-white hemolytic colonies | Positive | Positive |
| *S. simulans* | Non mannitol fermenter (pink colonies) | Round, convex, opaque white colonies (non hemolytic) | Positive | Negative |
| *S. sciuri* | Fermentation of mannitol (yellow colonies) | White to light grey colonies without any haemolytic activity | Positive | Negative |

**5-*Enterococcus faecalis***

|  | **Blood agar** | **Bile esculin test** | **Catalase test** | **Growth on tryptic soy broth with 6.5% NaCl** |
| --- | --- | --- | --- | --- |
| *E. faecalis* | Small, grayish-white, circular non-hemolytic colonies | Hydrolyze esculin in the presence of bile ( turns the entire slant dark brown to black) | Negative | Positive (turbidity) |
